# High-performance liquid chromatography – diode array detection method validation for amentoflavone-type biflavonoids in five Encephalartos species with potential neuroprotective activity

**DOI:** 10.1038/s41598-026-60998-6

**Published:** 2026-07-13

**Authors:** Zainab G. El-Natory, Hayam S. Ahmed, Dalia El Amir, Nada S. Abdelwahab, Abeer Moawad

**Affiliations:** 1 Pharmacognosy Department, Faculty of Pharmacy, Beni-Suef University, Beni-Suef, 62511 Egypt; 2 Pharmaceutical Analytical Chemistry Department, Faculty of Pharmacy, Beni-Suef University, Beni-Suef, 62511 Egypt

**Keywords:** *Encephalartos*, AChE, Antioxidant, HPLC, Total phenolic, Total flavonoid, Biochemistry, Biological techniques, Biotechnology, Chemical biology, Drug discovery, Plant sciences

## Abstract

*Ginkgo biloba* is a well-known food supplement for enhancing memory and is rich in biflavonoids. Biflavonoids are predominant in the Order Cycadales (cycads). Among cycads, five *Encephalartos* species, *E. ferox*,* E. kisambo*,* E. laurentianus*,* E. natalensis*, and *E. villosus*, were selected to assess the neuroprotective potential by estimation of the antioxidant and acetylcholinesterase (AChE) inhibition activities. *E. natalensis* and *E. ferox* strongly inhibited AChE (IC_50_=1.349 ± 0.041 and 1.948 ± 0.06 µg/mL respectively), while *E. kisambo* and *E. ferox* were the most potent antioxidant. Accordingly, *E. ferox* was selected for further investigation. Chromatographic isolation of the ethyl acetate fraction afforded amentoflavone (**1**), bilobetin (**2**), ginkgetin (**3**), naringenin (**6**), and apigenin(**7**). The isolated biflavonoids (**1–3**) showed potent AChE inhibitory activity with IC_50_= 2.146 ± 0.086, 0.762 ± 0.039, and 1.474 ± 0.061 µg/mL respectively compared to rivastigmine, the positive control (IC_50_= 3.357 ± 0.103 µg/mL). A validated high-performance liquid chromatography with diode array detection (HPLC-DAD) method was developed for the simultaneous estimation of five biflavonoids: amentoflavone, bilobetin, ginkgetin, isoginkgetin and sciadopitysin in the five *Encephalartos* species and *Ginkgo biloba.* In addition, total phenolic and total flavonoid contents were estimated in the selected plants. This study highlights *Encephalartos* as a potential source of bioactive compounds for further neuroactive drug discovery research.

**Supplementary Information:**

The online version contains supplementary material available at 10.1038/s41598-026-60998-6.

## Introduction

Alzheimer disease (AD) is a well-known degenerative disease distinguished by gradual loss of memory, disorientation, and impairment of nearly all intellectual functions. Furthermore, the risk of having AD increases at the age of 65 and doubles every five years^1^. Despite extensive study, little is known about treatments for slowing cognitive decline and the onset of dementia^1,2^. The pathogenesis of AD was explained by numerous hypotheses including the cholinergic theory^3^. Acetylcholine is a vital neurotransmitter responsible for facilitating cellular communication and is fundamental to memory retention and cognitive function. Any disruption in its transmission can lead to mental decline and behavioral shifts typically seen in Alzheimer’s patients. In a healthy brain, acetylcholinesterase (AChE) serves to break down acetylcholine. By inhibiting AChE, these substances help maintain higher levels of this essential messenger^4^. Many natural phytochemicals, including biflavonoids^5^, act as AChE inhibitors and may have neuroprotective potential.

Biflavonoids are flavonoid-flavonoid dimers with varied chemical structures due to the possibility of different flavonoid dimer combinations. For example, flavanone-flavone, flavone-flavone and flavone-flavanols are possible. In addition, the bond connections between the flavonoids may be C-C bond or C-O-C bond. Moreover, a connecting bond may have diverse positions: 3′-8′′, 3′-4′′′, 4′-4′′′, etc. In natural biflavonoids, many hydroxyl/methoxy groups are substituted at different positions^6^. The major occurrence of biflavonoids is in the gymnosperms. Their distribution and patterns of occurrence are very related to plant evolution^7^. Moreover, the different families of the gymnosperms have their unique pattern of biflavonoids, so they are regarded as excellent chemotaxonomic markers. Analysis of biflavonoids has been conducted since 1970 through thin layer chromatography (TLC) comparisons with relatively few reports employing advanced techniques^8^. Biflavonoids have diverse biological activities: antioxidant^9^, antimicrobial^10^, cytotoxic^11^, antitumor^12^, hepatoprotective^13^, and antimalarial^14^ activities. One of prominent gymnospermae plants is *Ginkgo biloba*. Its leaves are rich in biflavonoids to which much of its significant biological activity is attributed^15^. Ginkgetin and isoginkgetin demonstrated potential anti-Alzheimer’s properties through their ability to inhibit the acetylcholinesterase (AChE) enzyme^5^. Despite these findings, many gymnosperms are still unexplored concerning their phytochemicals and biological activities. A review of the Zamiaceae family revealed a significant knowledge gap within the genus *Encephalartos* as current literature only addresses a limited number of its species. Consequently, the current study focuses on a selection of five *Encephalartos* species: *E. ferox*, *E. natalensis*,* E. kisambo*,* E. villosus*, and *E. laurentianus* in addition to *Ginkgo biloba* for comparison.

*E. ferox* G. Bertol is a cycad member of the Zamiaceae family that is native to South Africa. Its leaves are utilized in traditional medicine for the prevention of various conditions, including the treatment of estrogen-dependent tumors and diabetes^16^. Scientific studies validated the antidiabetic and antioxidant effects of the leaves. Phytochemical study of the leaves revealed the presence of tannins, flavonoids, and alkaloids but no saponins or steroids^17^. Also, gas chromatograph–mass spectrometry (GC–MS) analysis of the fruit methanolic extract identified eight volatile compounds, in which *cis*-vaccenic acid and 9-octadecenoic acid 1,2,3-propanetriyl ester were the major detected metabolites^17,18^.

*E. villosus* Lem. is a decorative dwarf cycad often called the poor man’s cycad. Phytochemical study of the leaves revealed the presence of four flavone glycosides: luteolin 7-glucoside, luteolin-7-rutinoside, apigenin-7-glucoside, and luteolin-7-rhamnoside along with two illudalane sesquiterpenes: encephaldiene 1 and encephaldiene 2. The plant showed antifungal activity against *Aspergillus fumigatus* and antibacterial activity against *Streptococcus pneumoniae*, *Bacillus subtilis*, and *Escherichia coli*^19^. *E. villosus* Lem. demonstrated significant anti-inflammatory, antioxidant, and anti-apoptotic effects, potentially by suppressing the TLR4/NF-kB signaling pathway^20^.

*E. laurentianus* De Wild is reported to have cytotoxic, antioxidant, and antibacterial properties^21^. Silver Nanoparticles (AgNPs) synthesized using leaf extract exhibited antifungal properties both in vitro and in vivo experiments which was attributed to its content of flavonoids, flavonoid glycosides, phenolic, and organic acids^22^.

*E. natalensis* R.A.Dyer & I.Verd volatile emissions of the male and female cones were analyzed using GC–MS. An overall of 31 volatile compounds were detected, including 13 fatty acid derivatives, seven benzenoids, ten terpenoids, and one nitrogen-containing compound before and during receptivity and pollen release^23^.

*E. kisambo* Faden & Beentje, (synonym *E. voiensis*), is a large cycad species native to the Voi region of Kenya and possibly also grows in neighboring areas of Tanzania^24^. No literature was traced concerning its phytochemicals or biological studies.

Accordingly, we selected *E. ferox*,* E. natalensis*,* E. kisambo*,* E. villosus*, and *E. laurentianus* for the present study to investigate their chemical profiles applying different chromatographic and spectroscopic techniques. We also developed and validated RP-HPLC-DAD method for standardization of the selected plant species compared to *G. biloba* leaves. Amentoflavone and four of its methyl ether derivatives: bilobetin, ginkgetin, isoginkgetin, and sciadopitysin, were quantified in the five *Encephalartos* species and *Ginkgo biloba* leaves. In addition, acetylcholinesterase inhibition and antioxidant assays were analyzed for potential assessment of neuroactive potential. Furthermore, total phenolics and total flavonoids were performed for the six plants and correlated to the analytical study.

## Results and discussion

The antioxidant and acetylcholinesterase (AChE) inhibition assays were performed on five *Encephalartos* species, from which *E. ferox* showed a marked potency in both activities. Based on these results, *E. ferox* was selected for further chromatographic isolation of its phytochemicals. The isolated biflavonoids showed potent AChE inhibition. A validated HPLC was developed for estimation of five biflavonoids in *Encephalartos* species and *Ginkgo biloba*. Herein, we describe structural elucidation, HPLC analysis, and biological evaluation results.

### Identification of the isolated compounds

**Fig. 1:**
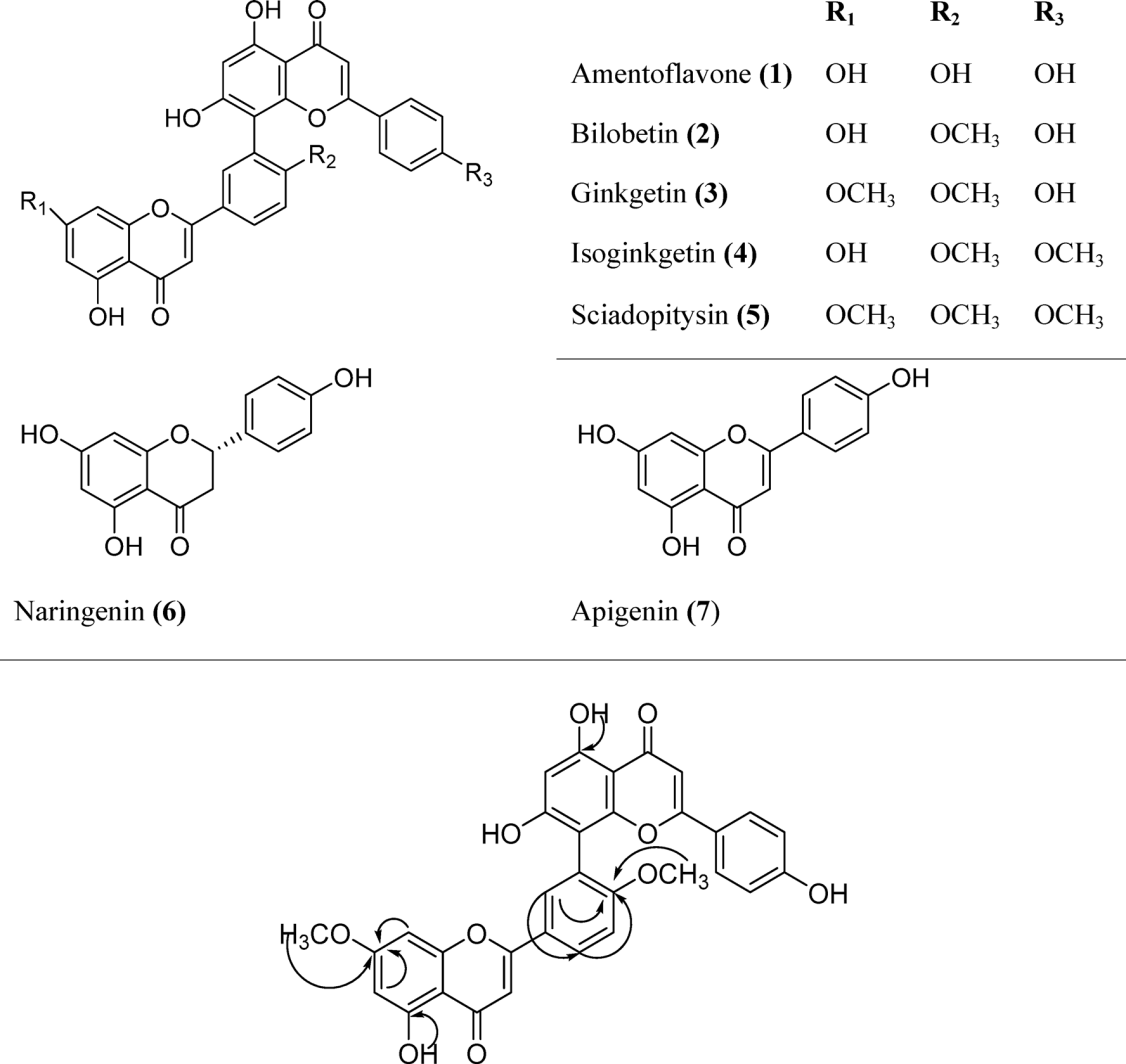
Chemical structures of the isolated compounds from *Encephalartos ferox* and HPLC analyzed amentoflavone-type biflavonoids with key HMBC correlations of ginkgetin (**3**).

Phytochemical investigation of the ethyl acetate extract of *E. ferox* leaflets afforded the isolation of five compounds. The structure of these compounds was elucidated through a combination of ^1^H-NMR, DEPTQ, and HMBC experiments and comparison with existing published data. The identified compounds were amentoflavone (**1**)^25^, bilobetin (**2**)^26^, ginkgetin (**3**)^27,28^, naringenin (**6**)^29^, and apigenin (**7**)^30^. Structure confirmation of ginkgetin (**3**) was done by extensive study of its HMBC data. Figure 1 illustrates the structures of the isolated compounds and key HMBC correlations of ginkgetin (**3**), while Table S_1_ (supplementary material) compiles the ^1^H-NMR data for the isolated flavonoids and biflavonoids.

Compound **6** was characterized by ^1^H-NMR spectroscopy in acetone-*d*_*6*_, and, to the best of our knowledge, these spectroscopic data are reported herein for the first time in this solvent: ^1^H-NMR (acetone-*d*_*6*_, 400 MHz): δ_H_ 12.17 (s, OH-5), 7.38 (d, *J* = 8.5 Hz, H-2′/H-6′), 6.89 (d, *J* = 8.5 Hz, H-3′/H-5′), 5.95 (d, *J =* 2.5 Hz, H-6/ H-8), 5.44 (dd, *J* = 12.8, 3.0 Hz, H-2), 3.17 (dd, *J* = 17.1, 12.8 Hz, H-3a), and 2.72 (dd, *J* = 17.1, 3.0 Hz, H-3b).

### High-performance liquid chromatography – diode array detection (HPLC-DAD) analysis

Five major biflavonoids of *Ginkgo biloba* leaves were analyzed in a previous study^31^. The five biflavonoids were amentoflavone (**1**), bilobetin (**2**) and ginkgetin (**3**), isoginkgetin (**4**), and sciadopitysin (**5**). The biflavonoids source was described under the “Material and Methods” section. A comparative analysis of amentoflavone-class biflavonoids was conducted across the five *Encephalartos* species and compared with *Ginkgo biloba*. All the five investigated compounds (Fig. 1) are characterized by 3′-8″ linkage between two apigenin units in which bilobetin is an amentoflavone 4′-monomethyl ether. Isoginkgetin and ginkgetin are dimethyl ether derivatives of amentoflavone; isoginkgetin is 4′,4‴ dimethyl ether, and ginkgetin is 7,4′ dimethyl ether. Meanwhile, sciadopitysin is 7,4′,4‴-trimethyl ether. The presence and location of these methoxy groups influence the chemical properties and polarity of the compounds, facilitating their separation using RP-C_18_ HPLC silica column with gradient elution, using the described method in the “Material and Methods” section. The HPLC method demonstrated effective compound separation with retention times varying from 22.4 to 32.9 min. The optimal detection wavelength for biflavonoids in a single analytical run was determined to be 330 nm.

**Fig. 2:**
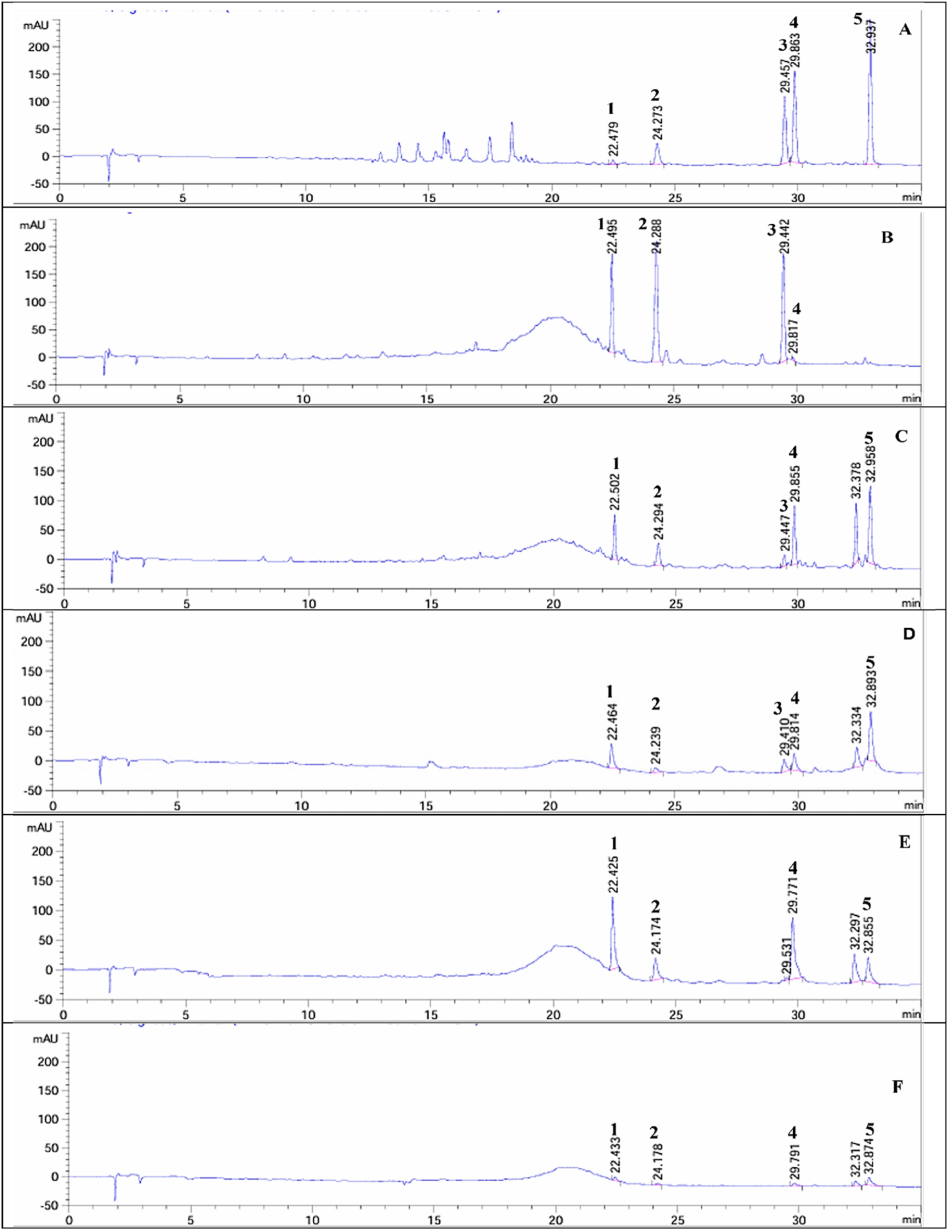
HPLC Chromatograms (330 nm) of the methanol extract of *Ginkgo biloba* (**A**), *Encephalartos ferox* (**B**), *E. natalensis* (**C**), *E. villosus* (**D**), *E. kisambo* (**E**), *E. laurentianus* (**F**). The peaks refer to: Amentoflavone (**1**), Bilobetin (**2**), Ginkgetin (**3**), Isoginkgetin (**4**), and Sciadopitysin (**5**).

After method development and achievement of complete separation between the studied components, the method was validated following the guidelines of the International Council for Harmonisation of technical requirements for pharmaceuticals for human use (ICH)^32^. Specificity was confirmed by complete separation of the five compounds in each extract as shown in Fig. 2; Table 1. The value of the resolution between the two adjacent compounds was more than 1.5, which confirmed complete separation between the adjacent components. To evaluate linearity, seven-point calibration curves were constructed for each compound within the defined concentration range. The regression analysis results, including slopes, intercept, and correlation coefficients, are presented in Table 1. All calibration curves exhibited linearity across the tested concentration ranges (2.5–50 µg/mL for components **1**, **2**, and **3** and 2.5–60 µg/mL for components **4** and **5**) with correlation coefficients (R^2^) varying between 0.9988 and 0.9998. This indicated a strong correlation and excellent linearity for the method. The method also exhibited good precision with intra-day relative standard deviation (RSD) values ranging from 2.28% to 4.17% and inter-day RSD values spanning 1.90% to 4.98%. These RSD values are within acceptable thresholds, indicating the method’s precision. The compounds exhibited average recovery rates between 97.06% and 103.88%, signifying good recovery of the respective samples and confirming the accuracy of the reported method. The low values of limit of detection (LOD) and limit of quantification (LOQ) shown in Table 1 confirmed the high sensitivity of the developed HPLC method.

**Table 1:** Regression and validation parameters of the developed HPLC method.

	Amentoflavone	Bilobetin	Ginkgetin	Isoginkgetin	Sciadopitysin
Regression parameters					
Linearity range	2.5–50 µg/mL			2.5–60 µg/mL	
Slope	48.3890	28.5700	30.4020	31.1590	9.8605
Intercept	1.9463	-13.8060	-23.6630	-24.8820	13.3690
Correlation coefficient (R^2^)	0.9988	0.9994	0.9997	0.9991	0.9998
Validation parameters					
Accuracy^ab^ (Mean±%RSD)	97.20 ± 4.21	103.88 ± 3.45	103.13 ± 3.69	102.24 ± 4.71	97.06 ± 5.30
Repeatability^ac^ (%RSD)	2.70	2.55	4.17	2.28	2.50
Intermediate^ad^ precision (%RSD)	4.49	4.19	4.49	1.90	4.98
LOD	0.70 µg/mL	0.78 µg/mL	0.75 µg/mL	0.78 µg/mL	0.83 µg/mL
LOQ	2.5 µg/mL				

^a^Average of three experiments.

^b^Mean% recovery of 8 different concentrations.

^c^Relative standard deviation of three concentrations of each component (5, 20 and 30 µg/mL) on the same day.

^d^Relative standard deviation of three concentrations of each component (5, 20 and 30 µg/mL) on three different days.

System suitability parameters were calculated to confirm the efficiency of the chromatographic system. All the separated peaks were nearly symmetric, which was confirmed by the calculated tailing factor. Additionally, complete separation was observed between all the separated components and plant extract matrices confirming the specificity of the developed approach. It also ensured by the acceptable values of both resolution (Rs) and selectivity (α) factors. Moreover, column efficiency was tested and confirmed by the high values of theoretical plates (N) and low value of height equivalent to a theoretical plate (HETP). All parameters were aligned with the reference value [USP]^33^, ensuring the validity of the chromatographic system presented in Table 2.

**Table 2:** Summary of parameters required for system suitability testing of the developed HPLC method.

Parameter	Amentoflavone	Bilobetin	Ginkgetin	Isoginkgetin	Sciadopitysin	Reference value*
Retention time (t_R_)	22.47	24.24	29.41	29.75	32.85	-------
Symmetry factor	1.01	1.01	1.02	1.02	1.01	1
Resolution (Rs)	6.00	15.42	1.71	10.67		≥ 1.5
Capacity factor (k′)	9.55	10.36	12.82	13.09	14.55	1–10
Selectivity (α)	1.08	1.19	1.02	1.12		≥ 1.0
N	215,296	62,500	164,295	96,100	467,856	*
HETP (mm)	0.0012	0.0040	0.0015	0.0026	0.0003	ʯ

N: number of theoretical plates.

HETP: height equivalent to a theoretical plate.

*: The higher the value, the better the column efficiency.

ʯ: The lower the value, the better the column efficiency.

The results revealed that amentoflavone (**1**) was the most abundant metabolite in *E. kisambo* (215.3 µg/g DW) and *E. ferox* (98.3 µg/gDW) (Table 3). In contrast, *E. laurentianus* exhibited the lowest concentration, while *Ginkgo biloba* leaves contained 25.1 µg/g DW. Bilobetin (**2**) was found in significant amounts in *E. ferox* (314.7 µg/g DW) and *E. kisambo* (114.4 µg/g) but at lower levels in *E. laurentianus*. *Ginkgo biloba* leaves showed 401.1 µg/g DW. Ginkgetin (**3**) was notably present in *E. ferox* (226.1 µg/g DW) and *E. villosus* (68.7 µg/g DW), with lower amounts in another species, and *Ginkgo biloba* provided 668.4 µg/g DW. Isoginkgetin (**4**) was significantly detected in *E. kisambo* (357.3 µg/g DW) and *E. natalensis* (112.2 µg/g DW), and trace amount in *E. ferox*, while *Ginkgo biloba* leaves contained 866.2 µg/g DW. Sciadopitysin (**5**) demonstrated substantial presence in *E. villosus* (745.8 µg/g DW) and *E. natalensis* (445.5 µg/g DW) but was not detected in *E. ferox*, and *G. biloba* yielded 2028.6 µg/g DW.

**Table 3:** Quantified amounts of the analyzed biflavonoids in different *Encephalartos* species and *Gingko biloba* leaves by RP- HPLC-DAD.

Concentration (µg/g DW) *±SD.	*E. ferox*	*E. natalensis*	*G. biloba*	*E. laurentianus*	*E. kisambo*	*E. villosus*
Amentoflavone (1)	98.3 ± 0.27	44.1 ± 0.05	25.1 ± 0.24	NQ	**215.3 ± 3.02**	61.7 ± 0.25
Bilobetin (2)	314.7 ± 0.89	63.8 ± 1.29	**401.1 ± 4.32**	13.6 ± 2.8	114.4 ± 1.48	33.4 ± 3.12
Ginkgetin (3)	226.1 ± 1.18	24.4 ± 0.35	**668.4 ± 0.21**	NQ	NQ	68.7 ± 0.37
Isoginkgetin (4)	NQ	112.2 ± 0.52	**866.2 ± 2.11**	13.3 ± 1.02	357.3 ± 1.71	44.2 ± 0.12
Sciadopitysin (5)	ND	445.5 ± 0.2 6	**2028.6 ± 2.08**	74.8 ± 1.39	409.6 ± 4.60	745.8 ± 3.15

*Average of three determinations.

NQ: Not Quantified ND: Not Detected.

The compositional profile of the analyzed compounds in *G. biloba* leaves showed a high degree of correlation with the findings of a previous report^31^ in the elution order of the main compounds but not in their specific concentrations.

### Statistical analysis of the results obtained from different plant samples

One-way analysis of variance (ANOVA) analysis demonstrated highly significant variations between the studied samples, in which the calculated F-values were substantially higher than the corresponding critical F-values (F > Fcrit) with extremely low P-values (*P* < 0.05). For example, *E. ferox* showed a highly significant difference among groups (F = 6918.042, *p* = 2.71 × 10^− 24^), indicating marked variability between the analyzed parameters. Similarly, significant differences were observed for the other investigated species/extracts, confirming the reliability and discriminatory power of the obtained results (Table S_3_).

## Biological study

### Acetylcholinesterase (AChE) inhibition

**Fig. 3:**
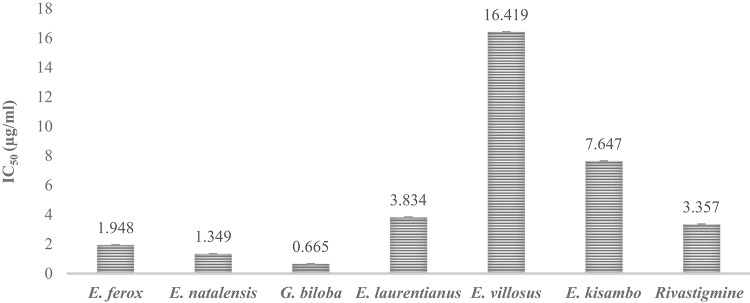
Inhibitory concentration of tested 80% methanolic extracts (IC_50_) against acetylcholinesterase (AChE).

Acetylcholinesterase (AChE) inhibition assay was performed on five *Encephalartos* species and *G. biloba* leaves using the positive control, rivastigmine (Fig. 3; Table 4). *G. biloba*,* E. natalensis*, and *E. ferox* showed marked inhibitory effect with IC_50_= 0.665 ± 0.02, 1.349 ± 0.041, and 1.948 ± 0.06 µg/mL respectively, which was more potent than the positive control, rivastigmine (3.357 ± 0.103 µg/mL). *E. laurentianus* was equally potent (3.834 ± 0.117 µg/mL); *E. kisambo* showed half the potency (7.647 ± 0.234 µg/mL); and *E. villosus* showed moderate activity 16.419 ± 0.502 µg/mL).

**Table 4:** Total phenolic content, total flavonoid content, antioxidant activity and acetylcholinesterase inhibition activity (IC_50_) of selected *Encephalartos* species and *Ginkgo biloba* leaves.

Plant name	TPC (mg GAE/g DW)	TFC (mg QE/g DW)	DPPH (mg AEAC/g DW)	Phosphomolybdate (mg AEAC/g DW)	AchE IC_50_ (µg/mL)
*E. ferox*	386.11 ± 1.91	61.37 ± 0.32	345.45±0.79	453.88±0.31	1.948 ± 0.06
*E. natalensis*	129.97 ± 1.109	24.55 ± 0.9	108.93±1.44	-	1.349 ± 0.041
*G. biloba*	166.57 ± 1.93	58.48 ± 2.25	121.66±2.01	119.63±0.84	0.665 ± 0.02
*E. laurentianus*	298.36 ± 2.12	40.23 ± 0.18	353.26±0.61	277.06±0.95	3.834 ± 0.117
*E. villosus*	59.21 ± 1.97	25.74 ± 1.22	101.61±1.18	-	16.419 ± 0.502
*E. kisambo*	593.70 ± 2.41	82.64 ± 0.32	360.32±1.48	903.25±0.846	7.647 ± 0.234
Amentoflavone (1)	-	-	-	-	2.146 **±** 0.086
Bilobetin (2)	-	-	-	-	0.762 ± 0.039
Ginkgetin (3)	-	-	-	-	1.474 ± 0.061
Rivastigmine	-	-	-	-	3.357 ± 0.103

Data represents Mean values ± standard deviation (*n* = 3) DW: Dry weight of plant material.

**TPC**: Total phenolic content was expressed as mg gallic acid equivalent per gram of dry weight (GAE/g DW).

**TFC**: Total flavonoid content was expressed as mg quercetin equivalent per gram of dry weight (mg QE/g DW).

Total antioxidant activity was expressed as mg ascorbic acid equivalent antioxidant capacity per gram of dry weight (AEAC/g DW).

The observed differences in AChE inhibition are likely related to variations in phytochemical composition and the relative abundance of bioactive constituents. Previous investigation of AChE inhibitory activity of amentoflavone, bilobetin, ginkgetin, isoginkgetin, sciadopitysin, and apigenin verified that ginkgetin and isoginkgetin significantly inhibited AChE, while the other compounds showed moderate activity^5^. The previous findings support our findings in which both monoflavonoids and biflavonoids from *G. biloba* leaves contribute to AChE inhibition, while ginkgolides exert negligible effects^34^. The strong inhibitory performance of the three extracts, particularly those exceeding rivastigmine, suggests their potential as promising natural cholinesterase inhibitors for alzheimer’s disease therapy.

The biological activities of the isolated compounds, amentoflavone, bilobetin, and ginkgetin, were evaluated, and the results were presented in Table 4. The tested compounds exhibited potent acetylcholinesterase (AChE) inhibitory activity with bilobetin showing the strongest effect (IC_50_= 0.762 ± 0.039 µg/mL), followed by ginkgetin (1.474 ± 0.061 µg/mL) and amentoflavone (2.146 ± 0.086 µg/mL). All tested compounds demonstrated significant inhibitory activity than the standard rivastigmine (IC_50_= 3.357 ± 0.103 µg/mL) under the experimental conditions employed. Interestingly, bilobetin was found to be the most active compound in the present study, whereas previous investigations of biflavonoids isolated from *Ginkgo biloba*^5^ reported comparatively lower AChE inhibitory activity for this compound. Such variation may be attributed to compound purity or variability in assay conditions. The remarkable activity of bilobetin observed herein suggests that it may represent one of the major contributors to the AChE inhibitory potential of the studied plant and highlights its promise as a natural lead compound for the development of anti-alzheimer’s agents.

### Antioxidant activity

The antioxidant properties of various *Encephalartos* species were primarily evaluated using 2,2-diphenyl-1-picrylhydrazyl assay (DPPH), which quantifies the ability of phenols to donate protons and react with DPPH. This study measured the total antioxidant capacity by assessing the cumulative ability of sample compounds to neutralize free radicals. Among the tested *Encephalartos species*, the *E. kisambo* extract exhibited the strongest scavenging activity, registering 360.32±1.48 mg AEAC/g. This was closely followed by *E. laurentianus* and *E. ferox* at values of 353.26±0.61 and 345.45±0.79 mg AEAC/g respectively. In contrast, *G. biloba* extract demonstrated a scavenging effect of 121.66±2.01 mg AEAC/g, while *E. natalensis* and *E. villosus* extracts showed the lowest scavenging effects at 108.93±1.44 mg and 101.61±1.18 mg AEAC/g respectively. These findings align with the TPC results. These phytochemicals likely contribute to the observed scavenging activity in this experiment. Correlation analysis indicated a strong positive relationship between DPPH free radical scavenging activity and TPC and a moderate correlation between DPPH assay and TFC. (Table 4)

The phosphomolybdate method provides a quantitative measure of total antioxidant capacity in terms of ascorbic acid equivalent. Among the tested extracts, *E. kisambo* demonstrated the highest antioxidant capacity (903.25±0.846 AEAC/g DW), followed by *E. ferox* and *E. laurentianus* (453.88±0.31 and 277.06±0.95 AEAC/g DW respectively). Meanwhile, *G. biloba* extract demonstrated a scavenging effect of 119.63±0.84 mg AEAC/g. These results are consistent with DPPH assay (Table 4). The potent antioxidant activity of *E. kisambo*, *E. ferox*, and *E. laurentianus* extracts suggests a significant presence of antioxidant phytochemicals within these samples, potentially due to high levels of phenolic compound.

These findings are consistent with the previous reports^17,21^, which demonstrated that the methanolic extract of *E. ferox* leaflets exhibits significant antioxidant activity, effectively scavenging ABTS and DPPH radicals with IC_50_ values of 68.3 and 308 µg/mL respectively. In addition, the crude leaf extract of *E. laurentianus* showed strong ABTS radical scavenging activity with a percentage inhibition of 82.6%.

### Total phenolic and total flavonoid contents

TPC of *E. kisambo*, *E. ferox*, and *E. laurentianus* were 593.70 ± 2.41, 386.11 ± 1.91, and 298.36 ± 2.12 mg GAE/g DW respectively. Meanwhile, *G. biloba* exhibited a TPC of 166.57±1.93 mg GAE/g DW, indicating that *E. kisambo*, *E. ferox*, and *E. laurentianus* possess higher phenolic concentrations than *G. biloba* (Table 4).

TFC of *E. kisambo* and *E. ferox* were 82.64 ± 0.32 and 61.37 ± 0.32 mg QE/g DW respectively. Meanwhile, *G. biloba* exhibited a TFC of 58.48 ± 2.25 mg QE/g DW, indicating that *E. kisambo* and *E. ferox* possess higher flavonoid concentrations than *G. biloba* leaves (Table 4).

Biflavonoids exhibit enhanced neuroprotective activity compared to single-unit flavonoids. In the context of Alzheimer’s disease, they are particularly effective at modulating amyloid-β (Aβ) aggregation by redirecting it away from toxic oligomers and fibrils toward non-toxic, unstructured forms. They also reduce the toxicity of existing Aβ oligomers more efficiently. Although their exact mechanisms are not fully understood, biflavonoids are believed to influence Aβ aggregation pathways and provide direct protection to neuronal cells, highlighting their potential therapeutic significance^35^.

## Conclusion

This study represents the first validated HPLC method for simultaneous analysis of five biflavonoids in five *Encephalartos* species. Many of the studied biflavonoids are reported for the first time in most of the studied *Encephalartos* species. Among the five *Encephalartos* species, *E. kisambo* leaflets had the highest amentoflavone and isoginkgetin contents than *Ginkgo biloba* leaves. *E. natalensis and E. ferox* showed marked AChE inhibitory effect. The descending order of the antioxidant activity was *E. kisambo > E. ferox > E. laurentianus >Ginkgo biloba > E. natalensis >E. villosus* which also align with TPC and TFC. Being more potent than *Ginkgo biloba* in AChE inhibition and antioxidant activity, many of studied *Encephalartos* species could be promising botanicals for inclusion in dietary supplements for management of Alzheimer’s disease and other neuronal related diseases. Among the isolated biflavonoids, bilobetin exhibited the strongest AChE inhibitory activity, surpassing the reference drug, rivastigmine, and highlighting its potential contribution to the AChE inhibition properties of the studied species. This study highlights *Encephalartos* as a potential source of bioactive compounds for further neuroactive drug discovery research.

## Materials and methods

### General experimental procedures

Thin layer chromatography (TLC) precoated normal phase silica gel plates GF254 (20 × 20 cm) (Merck), silica gel (230–400 mesh), and Sephadex LH-20 (Pharmacia Biotech, Uppsala), were used as stationary phases for chromatographic isolation of secondary metabolites. Vanillin-sulphuric acid (5%) spray reagent was used. Nuclear Magnetic Resonance (NMR) spectroscopy was employed to characterize the isolated compounds using a Bruker AVANCE III spectrometer. The NMR instrument is equipped with a BBFO Smart Probe and a Bruker 400 AEON Nitrogen-Free Magnet, operating at a proton (^1^H) frequency of 400.13 MHz. Spectra were recorded in DMSO-*d6* and acetone-*d6* with chemical shift values reported in parts per million (ppm) on the δ scale. Post-acquisition processing of the NMR data was performed using Topspin 3.1 software. RP-HPLC-DAD chromatographic analysis was performed using an HPLC system (Agilent Technologies 1260 Infinity) equipped with quaternary pump, auto-sampler, and DAD detector. HPLC- grade solvents: acetonitrile, methanol, double-distilled deionized water, and formic acid, were obtained from Merck (Germany), and analytical grade solvents: CHCl_3,_ ethyl acetate,methanol, dichloromethane, n-hexane, sulfuric acid, and vanillin, were obtained from Piochem (Egypt). UV–Visible spectrophotometer (Agilent 8453, Germany) was used for measurement of TPC, TFC, and antioxidant capacity. Chemicals for total phenolic (TPC), total flavonoid (TFC), and antioxidant assays, including aluminum chloride, ammonium molybdate, ascorbic acid, 2,2-diphenyl-1-picrylhydrazyl (DPPH), Folin–Ciocalteu reagent, gallic acid, quercetin, rutin, sodium carbonate, and sodium phosphate were purchased from Sigma-Aldrich (St. Louis, MO). Chemicals for biological study including the acetylcholinesterase (AChE) inhibitor screening assay kit (K197-100) were purchased from BioVision (USA).

### Plant material

Leaflets of *E. ferox* and *E. natalensis* were collected in December 2023 from cultivated specimens growing in El-Abd Garden on the Cairo-Alexandria Desert Road (68 km from Cairo) and identified by agronomist Rabea Sharway. *E. laurentianus* and *E. villosus* leaflets were collected on July 2025 from cultivated plants in El-Zohrya Garden, El-Zamalek, Cairo, Egypt. *E. kisambo* leaflets was obtained during the same period from cultivated stock at Cactus Helal Nursery, Al Mansourya, Giza, Egypt. Identification of the *Encephalartos* species was confirmed by Eng. Trease Labib, Former Head of El-Orman Botanical Garden, Giza, Egypt. *Ginkgo biloba* leaflets were acquired from Harraz Herbal Store, Cairo, Egypt, a licensed herbal supplier. All samples were obtained exclusively from cultivated plants, and no material was collected from wild populations, ensuring full alignment with the International Union for Conservation of Nature (IUCN) Policy Statement on Research Involving Species at Risk of Extinction (1989) which emphasizes the use of cultivated and non-lethal sampling strategies whenever feasible. No CITES-regulated wild specimens were involved^36^. Plant samples were shade-dried and powdered and stored at 4 °C until use.

Voucher specimens were deposited in the Pharmacognosy Department Herbarium, Faculty of Pharmacy, Beni-Suef University under the accession numbers BUPD-26-01 for *E. ferox*, BUPD-26-02 for *E. natalensis*, BUPD-26-03 for *E. kisambo*, BUPD-26-04 for *E. villosus*, BUPD-26-05 for *E. laurentianus*, and BUPD-26-06 for *Ginkgo biloba*, in which they are publicly accessible upon request. All collection and handling procedures comply with institutional, national and international regulations, including IUCN guidelines for responsible scientific collection.

### Biflavonoids source

Amentoflavone, bilobetin, and ginkgetin were isolated in this work from *E. ferox*, while isoginkgetin was previously isolated from *Dioon edule* leaves^37^ and sciadopitysin from *Dioon spinolsum* leaves^38^ in our previous communications.

### Extraction and isolation

#### Extraction and isolation of Encephalartos ferox flavonoids and biflavonoids

The powdered leaflets of *E. ferox* (0.5 kg) were extracted with ethyl acetate by maceration then filtered through Whatman No.1 filter paper. The solvent evaporated under reduced pressure at 40˚C. The crude extract (40 g) was suspended in distilled H_2_O and defatted with *n*-hexane to yield 30 g defatted ethyl acetate biflavonoid-rich fraction (BFRF). This BFRF was subjected to chromatographic isolation of biflavonoids.

**Fig. 4:**
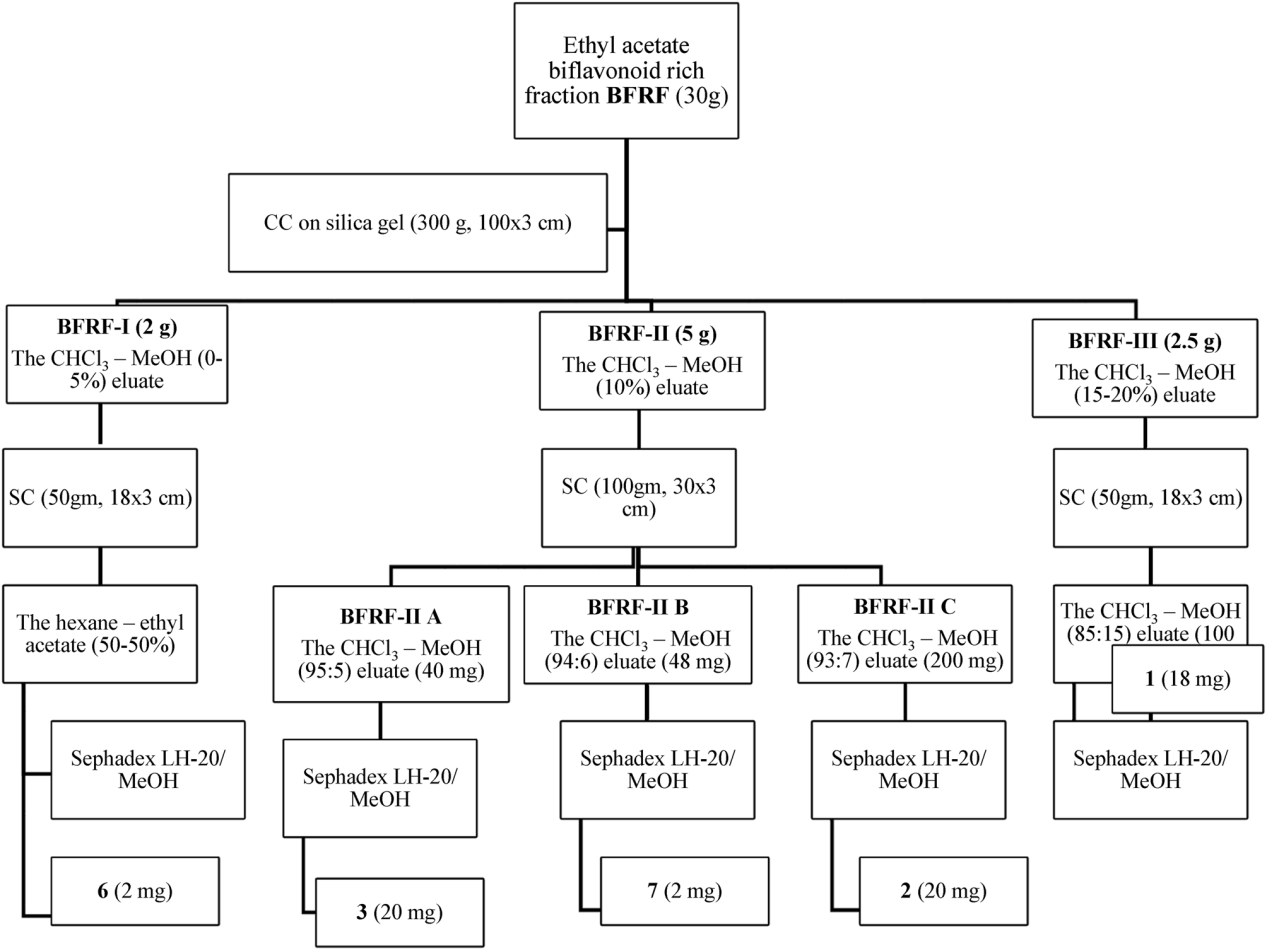
Scheme of the isolation of the main components from *Encephalartos ferox* leaflets.

The ethyl acetate biflavonoid-rich fraction (BFRF) of *E. ferox* leaflets was subjected to chromatographic isolation of flavonoids and biflavonoids (Fig. 4). BFRF (30 g) was subjected to silica gel column chromatography (CC) (300 g, 100 × 3 cm) and eluted with gradient CHCl_3_–MeOH eluent (0–50% in 5% increments, then 50–100% in 10% increments). Fractions (100 mL each) were collected and monitored with TLC using solvent system CHCl_3_–MeOH (9.5:0.5 to 7:3) and spraying with vanillin/H_2_SO_4_ spray reagent then heating. Similar fractions were pooled together to yield three subfractions (BFRF-I to BFRF-III). BFRF-I (2 g) was fractionated on CC (50 g, 18 × 3 cm) with gradient *n*-hexane-ethyl acetate (10% increments). The fraction eluted with 50% EtOAc in hexane was chromatographed using Sephadex LH-20 eluted with MeOH to yield 2 mg of compound (**6**).

BFRF-II (5 g) was fractionated on silica gel column (100 g, 30 × 3 cm) eluted with CHCl_3_–MeOH in 1% increments of MeOH to give three subfractions (BFRF-IIA-C). BFRF-II A (40 mg) was purified using Sephadex LH-20 eluted with MeOH to yield 20 mg of compound **(3)**. BFRF-II B (48 mg) was purified using Sephadex LH-20 eluted with MeOH to yield 2 mg of compound **(7)**. BFRF-II C (200 mg) was similarly purified to yield 20 mg of compound **(2)**.

BFRF-III (2.5 g) was fractionated on CC (50 g, 18 × 3 cm) with 1% increments of CHCl_3_ to give one subfraction. The CHCl_3_–MeOH (85:15) eluate (100 mg) was purified using Sephadex LH-20 eluted with MeOH to yield 18 mg of compound **(1)**. Isolation scheme was represented in Fig. 4.

#### Extraction for TPC, TFC, antioxidant assays, and acetylcholinesterase inhibition activity

The six plant leaflets under investigation (0.25 g each) were separately subjected to extraction with 50 mL of 80% methanol for 2 h at room temperature on an orbital shaker set at 200 rpm^39^. The plant powder was re-extracted under identical conditions for another 30 min, filtered to 100 mL volumetric flasks, and diluted to their final volume with 80% methanol. Extracts were kept in refrigerator till use.

### Extraction for RP-HPLC-DAD analysis

Different amounts of powdered leaflets of different *Encephalartos* species and *Ginkgo biloba* were separately extracted with methanol using ultra-sonication for 10 min (3 × 3.0 mL). The extract was filtered, and the volume of each extract was completed to 10 mL with methanol. Finally, solutions were filtered through a 0.45-µm membrane filter before injection into HPLC instrument. Each extraction was injected in triplicate (Table 2 supplementary).

### Acetylcholinesterase inhibition activity

Acetylcholinesterase inhibitory activity was evaluated using a commercial AChE inhibitor screening kit (BioVision, K197-100) according to the manufacturer’s instructions^40^. Test samples were added to wells along with inhibitor control (rivastigmine), solvent control, and enzyme control (EC), while assay buffer was added to background control (BC) wells. Diluted AChE enzyme was then added to all wells except [BC], and the volume was adjusted with assay buffer. After incubation at room temperature for 10–15 min, the reaction was initiated by adding a freshly prepared mixture of AChE substrate and probe reagent. Absorbance was recorded kinetically at 412 nm, and AChE inhibition was calculated as percentage inhibition relative to the enzyme control after background subtraction.

### Antioxidant activity evaluation

#### DPPH (2, 2-diphenyl-1-picrylhydrazyl) free radical scavenging assay

DPPH assay was evaluated according to Bhatti et al. (2015) with slight modification^41^. Briefly, 50 µL of the sample solution was mixed with 950 µL of methanolic DPPH solution (3.4 mg/100 mL) and incubated at 37 °C for one hour in the dark. Absorbance was measured at 517 nm using a UV–Visible spectrophotometer (Agilent 8453, Germany). Ascorbic acid was used as a positive control. The scavenging activity was calculated using the standard equation, and the antioxidant capacity was expressed as mg ascorbic acid equivalent antioxidant capacity per gram dry weight (mg AEAC/g DW).

### Phosphomolybdenum assay

Total antioxidant capacity was determined using the phosphomolybdenum method according to Prieto et al.^42^. Ascorbic acid was used as a positive control. The antioxidant activity was calculated using the standard equation and expressed as mg ascorbic acid equivalent antioxidant capacity per gram dry weight (mg AEAC/g DW).

### Total phenolic content (TPC)

TPC was determined using the Folin–Ciocalteu method according to Bhatti et al.^41^. Briefly, 0.1 mL of each extract solution (2.5 mg/mL) was mixed with diluted Folin–Ciocalteu reagent followed by sodium carbonate solution. After incubation at room temperature, the absorbance was measured at 725 nm using a UV–Visible spectrophotometer (Agilent 8453, Germany). Gallic acid was used for calibration, and the results were expressed as mg gallic acid equivalents per gram dry weight (mg GAE/g DW).

### Total flavonoid content

The aluminum chloride colorimetric method was used to spectrophotometrically determine the TPC in the plant extracts^43^. Each sample (exactly 0.5 ml) was combined with 0.5 ml of 2% ethanolic solution of aluminum chloride and incubated for one hour at room temperature. Then the absorbance was measured at 420 nm, and the standard curve was done using quercetin. TFC values were presented as quercetin equivalent per gram of dry sample weight (mg QE/g DW).

### RP-HPLC-DAD analysis

#### HPLC conditions

Quantitative analysis of five biflavonoids was performed using an Agilent 1260 Infinity LC system equipped with a diode array detector (DAD) (Agilent Technologies, Waldbronn, Germany). A 5 μm Fortis universal HS C18 column (size: 150 × 4.6 mm, 5 μm; Fortis Technologies Ltd., Cheshire, UK) was utilized for compound separation. The chromatographic conditions included a consistent column temperature of 30 °C and a flow rate of 1 mL/min for the 35-minute analysis. A 20 µL injection volume was used with detection at 330 nm. The mobile phase involved a gradient elution, comprising 0.1% formic acid in water (solvent A) and acetonitrile (solvent B) following the published method^31^ with some modification in the elution program. The used gradient program was set as follows: 0–5 min 2–10% B in A; 5–10 min, 10–30% B in A; 10–20 min, 30–50% B in A; 20–25 min, 50% B in A; 25–30 min, 80% B in A; and 30–35 min, 80–98% B in A.

#### Preparation of standard solutions

Individual standard stock solutions of five biflavonoids were prepared in methanol at a concentration of 100 µg/mL. From these stock solutions, various dilutions in the concentrations range of 2.5 µg/mL to 60 µg/mL were obtained by diluting appropriate aliquots with methanol. These diluted solutions were subsequently used for the construction of calibration curves. All the prepared solutions were stored at 20 °C until analysis. Before injection to HPLC instrument, all solutions were passed through a 0.45-µm syringe filter. The calibration functions for biflavonoids were subsequently computed based on their concentrations and the corresponding peak areas.

#### Method validation

The analytical method underwent validation in accordance with ICH^32^, ensuring its compliance with both national and international regulations. This adherence enhances the method’s reliability and broadens its applicability. The key validation parameters evaluated were selectivity, linearity, intraday precision (repeatability), intermediate precision, and accuracy. Selectivity was confirmed by comparing the chromatogram of the sample with those of the diluent and standard solutions and by differentiating between individuals of *G. biloba* and five species of *Encephalartos*. Linearity was tested by analyzing the previously prepared standard dilutions, following the previously mentioned chromatographic conditions. Calibration curves were then constructed from which regression equations were computed, and the correlation coefficients were calculated. Repeatability was expressed as % relative standard deviation (%RSD) and evaluated by analyzing three concentrations (5, 20, and 30 µg/mL) in triplicate on the same day. For intermediate precision, analysis of the three samples was repeated on a separate day, and the recovery data from both days were compared using %RSD. Accuracy was established at eight concentration levels by analysis of different pure samples and subsequent calculation of mean recovery percentages. The limits of detection and quantification were derived from the slope of the calibration curves established during calculation of the LOD, LOQ, and SD of the intercept. These limits were calculated using the formulas: LOD = 3.3 × σ/S and LOQ = 10 × σ/S, in which ‘S’ denotes the mean slope of the calibration curves, and ‘σ’ represents the standard deviation of the y-intercepts from the three linear calibration curves.

### Statistical analysis

One-way analysis of variance (ANOVA) was performed to evaluate the statistical significance of differences among the investigated groups.

## Supplementary Information

Below is the link to the electronic supplementary material.

Supplementary Material

## Data Availability

No datasets were generated or analysed during the current study.
