# Supplementary material for: High-performance liquid chromatography – diode array detection method validation for amentoflavone-type biflavonoids in five Encephalartos species with potential neuroprotective activity
Source: Sci Rep. 2026 Jul 13;16:21824. doi: 10.1038/s41598-026-60998-6 (PMC13365601; doi:10.1038/s41598-026-60998-6)
Supplement: Supplementary file 1 — Supplementary Material [file 41598_2026_60998_MOESM1_ESM.docx]

**Supplementary Material**

**High‑Performance Liquid Chromatography – Diode Array Detection**

**Method Validation for Amentoflavone-Type Biflavonoids in Five *Encephalartos* Species with potential neuroprotective activity**

 Zainab G. El-Natory^1+^, Hayam S. Ahmed^1+^, Dalia El Amir^1^, Nada S. Abdelwahab^2^, Abeer Moawad*^1^

*^1^Pharmacognosy Department, Faculty of Pharmacy, Beni-Suef University, Beni-Suef, 62511, Egypt.*

*^2^ Pharmaceutical Analytical Chemistry Department, Faculty of Pharmacy, Beni-Suef University, Beni-Suef, 62511, Egypt.*

*+ Authors have equal contributions*

*Corresponding author

**Abeer Moawad**

Pharmacognosy Department

Faculty of Pharmacy

Beni-Suef University

Beni-Suef, 62511, Egypt.

<Tel:+201148895396>

Email: [abeer.moawad@pharm.bsu.edu.eg](mailto:abeer.moawad@pharm.bsu.edu.eg), [Abeermoawad77@gmail.com](mailto:Abeermoawad77@gmail.com)

<https://orcid.org/0000-0002-0228-6757>

Figure S_1_: ^1^H NMR spectrum of amentoflavone (1) (400 MHz, DMSO-*d_6_*).

Figure S_2_: Expanded regions of ^1^H NMR spectrum of amentoflavone (1) (400 MHz, DMSO-*d_6_*).

Figure S_3_: ^1^H NMR spectrum of bilobetin (2) (400 MHz, DMSO-*d_6_*).

Figure S_4_: Expanded regions of ^1^H NMR spectrum of bilobetin (2) (400 MHz, DMSO-*d_6_*).

Figure S_5_: ^1^H NMR spectrum of ginkgetin (3) (400 MHz, DMSO-*d_6_*).

Figure S_6_: Expanded regions of ^1^H NMR spectrum of ginkgetin (3) (400 MHz, DMSO-*d_6_*).

Figure S_7_: DEPT-Q spectrum of ginkgetin (3) (100 MHz, DMSO-*d_6_*).

Figure S_8_: Expanded regions of DEPT-Q spectrum of ginkgetin (3) (100 MHz, DMSO-*d_6_*).

Figure S_9_: HMBC spectrum of ginkgetin (3) (400 MHz, DMSO-*d_6_*).

Figure S_10_: Expanded regions of HMBC spectrum of ginkgetin (3) (400 MHz, DMSO-*d_6_*).

Figure S_11_: Expanded regions of HMBC spectrum of ginkgetin (3) (400 MHz, DMSO-*d_6_*).

Figure S_12_: ^1^H NMR spectrum of naringenin (6) (400 MHz, acetone-*d_6_*).

Figure S_13_: Expanded regions of ^1^H NMR spectrum of naringenin (6) (400 MHz, acetone-*d_6_*).

Figure S_14_: ^1^H NMR spectrum of apigenin (7) (400 MHz, DMSO-*d_6_*).

Figure S_15_: Expanded regions of ^1^H NMR spectrum of apigenin (7) (400 MHz, DMSO-*d_6_*).

Table S_1_: ‎ ^1^H NMR (400 MHz) spectroscopic data of the isolated compounds from *Encephalartos ferox* leaflets.

Table S_2_. Extract Concentrations of Different Plant Species Used for HPLC Calibration.

Table S_3_: One Way ANOVA test for the results obtained from different plant samples

|   Figure S_1_: ^1^H NMR spectrum of amentoflavone **(1)** (400 MHz, DMSO-*d_6_*).    Figure S_2_: Expanded regions of ^1^H NMR spectrum of amentoflavone **(1)** (400 MHz, DMSO-*d_6_*). |
| --- |
|   Figure S_3_: ^1^H NMR spectrum of bilobetin **(2)** (400 MHz, DMSO-*d_6_*).    Figure S_4_: Expanded regions of ^1^H NMR spectrum of bilobetin **(2)** (400 MHz, DMSO-*d_6_*). |
|   Figure S_5_: ^1^H NMR spectrum of ginkgetin **(3)** (400 MHz, DMSO-*d_6_*).  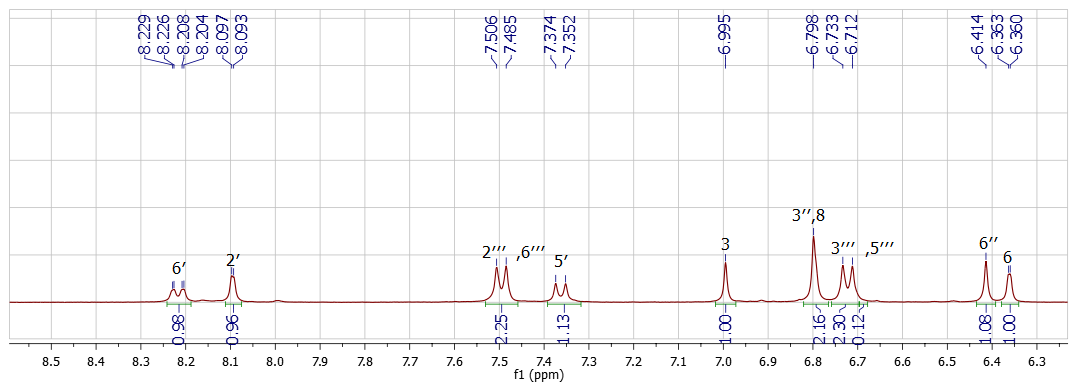  Figure S_6_: Expanded regions of ^1^H NMR spectrum of ginkgetin **(3)** (400 MHz, DMSO-*d_6_*).    Figure S_7_: DEPT-Q spectrum of ginkgetin **(3)** (100 MHz, DMSO-*d_6_*).       Figure S_8_: Expanded regions of DEPT-Q spectrum of ginkgetin **(3)** (100 MHz, DMSO-*d_6_*).    Figure S_9_: HMBC spectrum of ginkgetin **(3)** (400 MHz, DMSO-*d_6_*).    Figure S_10_: Expanded regions of HMBC spectrum of ginkgetin **(3)** (400 MHz, DMSO-*d_6_*).     Figure S_11_: Expanded regions of HMBC spectrum of ginkgetin **(3)** (400 MHz, DMSO-*d_6_*). |
|   Figure S_12_: ^1^H NMR spectrum of naringenin **(6)** (400 MHz, acetone-*d_6_*).     Figure S_13_: Expanded regions of ^1^H NMR spectrum of naringenin **(6)** (400 MHz, acetone-*d_6_*). |
|   Figure S_14_: ^1^H NMR spectrum of apigenin **(7)** (400 MHz, DMSO-*d_6_*).    Figure S_15_: Expanded regions of ^1^H NMR spectrum of apigenin **(7)** (400 MHz, DMSO-*d_6_*). |
|  |

**Table S_1_**: ‎ ^1^H NMR (400 MHz) spectroscopic data of the isolated compounds from *Encephalartos ferox* leaflets.

|  | **Compound Code** | | | | |
| --- | --- | --- | --- | --- | --- |
| **Position** | **1** | **2** | **3** | **6** | **7** |
| **2** | - | - | - | 5.44 (dd, *J*= ‎12.8, 2.96) | - |
| **3** | ‎‎6.81‎‎ | ‎6.90 | 6.99 | 2.72, eq. (dd, *J*= ‎17.1, 3)  3.17, ax. (dd, *J*= ‎17.1, 12.8) | ‎6.75‎ |
| **6** | ‎6.19 ‎‎(d, J = 1) | ‎6.20 | 6.36 (d, *J*= 1.44) | 5.95 (d, *J=* 2.5) | ‎6.17‎ |
| **‎8‎** | ‎6.45‎ (d, *J*= 1.‎‎4)‎ | ‎6.48‎ | ‎6.80‎ | ‎5.95 ( ‎d, *J*= 2.5)‎ | ‎6.47‎ |
| **‎2′‎** | ‎8.02‎ | ‎8.06‎ | ‎8.09 ‎‎(d, *J*= 1.72)‎ | ‎7.38 (d, *J*= 8.‎‎5)‎ | ‎7.91(d, *J*= 8.‎‎8)‎ |
| **‎3′‎** | ‎-‎ | ‎-‎ | ‎-‎ | ‎6.89 (d, *J*= ‎‎8.50)‎ | ‎6.93 ‎‎(d, *J*=8.44)‎ |
| **‎5′‎** | ‎‎7.13 ‎‎‎(d, *J*= 8.56)‎ | ‎7.34‎ ‎‎(d, *J*= 8.7‎‎6)‎ | ‎7.36‎ ‎‎(d, *J*= 8.84)‎ | ‎6.89 (d, *J*= ‎‎8.50‎)‎ | ‎6.93 ‎‎(d, *J*=8.44)‎ |
| **‎6′‎** | ‎7.99 ‎‎(d, *J*= 8.72)‎ | ‎‎8.16 ‎‎(d, *J*= 8.2‎‎)‎ | ‎8.22 ‎‎(dd, *J*= 1.2,8.‎‎84)‎ | ‎7.38 (d, *J*= 8.‎‎5)‎ | ‎7.91 (d, *J*=8.‎‎8)‎ |
| **3′′** | ‎‎ 6.77‎ | ‎6.78 | 6.80 | - | ‎- |
| **6′′** | ‎‎6.37‎ | ‎6.40 | 6.41 | - | ‎- |
| **8′′** | ‎- | ‎ | - | - | - |
| **3′′′ , 5′′′** | ‎6.70 ‎(d*, J*= 8.6) | ‎6.72‎ (d, *J*= 8.16) | 6.72 (d, *J*= 8.52) | - | - |
| **2′′′ , 6′′′** | ‎7.58 ‎‎‎(d, *J*= 8.56) | ‎‎7.50 (d, *J*= 8.24) | 7.50 (d, *J*= 8.52) | - | - |
| **4′-OCH_3_** | - | 3.78‎ | 3.79 | - | - |
| **7-OCH3** | - | - | 3.83 | - | - |
| **‎5-OH** | ‎12.98 (s)‎ | ‎12.92 (s)‎ | ‎12.91 (s)‎ | 12.17 (s) | 12.95 (s) |
| **‎5′′-OH** | ‎13.11 (s)‎ | ‎13.09 (s)‎ | ‎13.09 (s)‎ |  |  |

δ_H_ multi (*J* in Hz)

Compounds **1, 2, 3** and **7** are in DMSO-*d_6_* while compound **6** is in) acetone-*d_6_*(. ‎

Chemical shift values in ppm and *J* values (in Hz) are presented in parentheses.

**Table S_2_**. Extract Concentrations of Different Plant Species Used for HPLC Calibration.

|  | **Amount Used (g)** | | | | | |
| --- | --- | --- | --- | --- | --- | --- |
|  | *E. ferox* | *E. natalensis* | *G. biloba* | *E. laurentianus* | *E. villosus* | *E. kisampo* |
| Amentoflavone **(1)** | **2** | **2** | **2** | **2** | **1** | **1** |
| Bilobetin **(2)** | **2** | **2** | **1** | **2** | **1** | **1** |
| Ginkgetin **(3)** | **2** | **2** | **0.5** | **1** | **1** | **1** |
| Isoginkgetin **(4)** | **2** | **2** | **0.5** | **2** | **1** | **1** |
| Sciadopytsin **(5)** | **2** | **1** | **0.25** | **1** | **0.5** | **1** |

**Table S_3_:** One Way ANOVA test for the results obtained from different plant samples

| ***Encephalartos ferox*** | | | | | | |
| --- | --- | --- | --- | --- | --- | --- |
| ***Source of Variation*** | ***SS*** | ***df*** | ***MS*** | ***F*** | ***P-value*** | ***F crit*** |
| **Between Groups** | 312382.6 | 4 | 78095.66 | **6918.042** | **2.71E-24** | **3.055568** |
| **Within Groups** | 169.3304 | 15 | 11.28869 |  |  |  |
| **Total** | 312552 | 19 |  |  |  |  |
| ***Encephalartos natalensis*** | | | | | | |
| ***Source of Variation*** | ***SS*** | ***df*** | ***MS*** | ***F*** | ***P-value*** | ***F crit*** |
| **Between Groups** | 279707.9 | 4 | 69926.98 | **5401.14** | **8.69E-21** | **3.179117** |
| **Within Groups** | 168.3072 | 13 | 12.94671 |  |  |  |
| **Total** | 279876.2 | 17 |  |  |  |  |
| ***Gingko biloba*** | | | | | | |
| ***Source of Variation*** | ***SS*** | ***df*** | ***MS*** | ***F*** | ***P-value*** | ***F crit*** |
| **Between Groups** | 279707.9 | 4 | 69926.98 | **5401.14** | **8.69E-21** | **3.179117** |
| **Within Groups** | 168.3072 | 13 | 12.94671 |  |  |  |
| **Total** | 279876.2 | 17 |  |  |  |  |
| ***Encephalartos laurentianus*** | | | | | | |
| ***Source of Variation*** | ***SS*** | ***df*** | ***MS*** | ***F*** | ***P-value*** | ***F crit*** |
| **Between Groups** | 279707.9 | 4 | 69926.98 | **5401.14** | **8.69E-21** | **3.179117** |
| **Within Groups** | 168.3072 | 13 | 12.94671 |  |  |  |
| **Total** | 279876.2 | 17 |  |  |  |  |
| ***Encephalartos kisambo*** | | | | | | |
| ***Source of Variation*** | ***SS*** | ***df*** | ***MS*** | ***F*** | ***P-value*** | ***F crit*** |
| **Between Groups** | 279707.9 | 4 | 69926.98 | **5401.14** | **8.69E-21** | **3.179117** |
| **Within Groups** | 168.3072 | 13 | 12.94671 |  |  |  |
| **Total** | 279876.2 | 17 |  |  |  |  |
| ***Encephalartos villosus*** | | | | | | |
| ***Source of Variation*** | ***SS*** | ***df*** | ***MS*** | ***F*** | ***P-value*** | ***F crit*** |
| **Between Groups** | 279707.9 | 4 | 69926.98 | **5401.14** | **8.69E-21** | **3.179117** |
| **Within Groups** | 168.3072 | 13 | 12.94671 |  |  |  |
| **Total** | 279876.2 | 17 |  |  |  |  |
